# Efficacy of Endoscopic and Surgical Treatments for Gastroesophageal Reflux Disease: A Systematic Review and Network Meta-Analysis

**DOI:** 10.3390/jpm12040621

**Published:** 2022-04-12

**Authors:** Eun Jeong Gong, Chan Hyuk Park, Da Hyun Jung, Sun Hyung Kang, Ju Yup Lee, Hyun Lim, Do Hoon Kim, Endoscopic Therapy and Instrument Research Group under the Korean Society of Neurogastroenterology and Motility

**Affiliations:** 1 Department of Internal Medicine, Hallym University College of Medicine, Chuncheon 24253, Korea; gong-eun@hanmail.net; 2 Department of Internal Medicine, Hanyang University Guri Hospital, Hanyang University College of Medicine, Guri 11923, Korea; yesable7@gmail.com; 3 Division of Gastroenterology, Department of Internal Medicine, Severance Hospital, Yonsei University College of Medicine, Seoul 03722, Korea; jungdh@yuhs.ac; 4 Department of Internal Medicine, Chungnam National University School of Medicine, Daejeon 35015, Korea; porrtos@daum.net; 5 Department of Internal Medicine, Keimyung University School of Medicine, Daegu 42601, Korea; leejygi@naver.com; 6 Department of Internal Medicine, Hallym University Sacred Heart Hospital, Hallym University College of Medicine, Anyang 14068, Korea; hlim77@hallym.or.kr; 7 Department of Gastroenterology, University of Ulsan College of Medicine, Asan Medical Center, Seoul 05505, Korea

**Keywords:** gastroesophageal reflux disease, proton pump inhibitor, radiofrequency, plication, fundoplication

## Abstract

Although various endoscopic and surgical procedures are available for the treatment of gastroesophageal reflux disease (GERD), the comparative efficacy of these treatments has not been fully elucidated. This study aimed to comprehensively evaluate the efficacy of various endoscopic and surgical treatments for GERD. All relevant randomized controlled trials published through August 2021 that compared the efficacy of endoscopic and surgical GERD treatments, including radiofrequency energy delivery, endoscopic plication, reinforcement of the lower esophageal sphincter (LES), and surgical fundoplication, were searched. A network meta-analysis was performed to analyze treatment outcomes, including the requirement of proton pump inhibitor (PPI) continuation and GERD-health-related quality of life questionnaire score (GERD-HRQL). As such, 25 studies with 2854 patients were included in the analysis. Endoscopic plication, reinforcement of the LES, and surgical fundoplication were effective in reducing the requirement of PPI continuation compared to PPI therapy (pooled risk ratio (RR) (95% confidence interval [CI]): endoscopic plication, 0.34 (0.21–0.56); reinforcement of LES, 0.32 (0.16–0.63), and surgical fundoplication, 0.16 (0.06–0.42)). Radiofrequency energy delivery tended to reduce the requirement of PPI continuation compared to PPI therapy (RR (95% CI): 0.55 (0.25–1.18)). In terms of GERD-HRQL, all endoscopic and surgical treatments were superior to PPI therapy. In conclusion, all endoscopic or surgical treatments, except radiofrequency energy delivery, were effective for discontinuation of PPI medication, especially surgical fundoplication. Quality of life, measured by GERD-HRQL, also improved in patients who underwent endoscopic or surgical treatment compared to those who received PPI therapy.

## 1. Introduction

Gastroesophageal reflux disease (GERD) is a globally widespread disease, and its prevalence has increased [1]. Although lifestyle modifications, including weight reduction, may be recommended for relieving GERD symptoms, acid-suppressive medications, including proton pump inhibitors (PPIs), are the mainstay of treatment for GERD [2]. The administration of PPIs for 4 weeks can achieve approximately 70% healing rate and 60–90% complete symptom relief in patients with erosive esophagitis [3]. Current guidelines strongly recommend 4–8 weeks of PPI therapy as the initial treatment for GERD [2,4].

Nevertheless, pharmaceutical treatment for GERD has several limitations in clinical practice. First, GERD symptoms are likely to recur if acid-suppressive medication is discontinued [5]. Therefore, long-term medication is required for patients with PPI-dependent GERD [6]. The economic burden of long-term medication may be a concern [7]. Second, 10–40% of patients with GERD do not respond to PPI therapy [8,9]. Refractory GERD impairs patients’ quality of life and work productivity despite acid-suppressive medications [10,11].

Representative non-pharmaceutical treatments for GERD include surgical fundoplication, such as Nissen fundoplication, which was first introduced in 1955 by Rudolph Nissen [12]. Since 1991, Nissen fundoplication has been performed laparoscopically [13]. A multicenter randomized controlled trial (RCT) in Europe revealed that laparoscopic anti-reflux surgery had comparable efficacy to that of PPI [14]. More recently, less-invasive therapeutic procedures for GERD have been developed, including radiofrequency energy delivery (Stretta^®^), endoscopic plication (EndoCinch^®^, NDO plicator^®^, EsophyX^®^, GERD-X^®^), and reinforcement of the lower esophageal sphincter (LES) (Enteryx^®^, Gatekeeper^®^, LINX^®^) [15,16]. Stretta^®^ is performed transorally using a commercially available device guided by endoscopy, and is applied to the distal esophagus, esophagogastric junction, and cardia of the stomach [17]. Low-power radiofrequency energy delivered to the muscular layer of the esophagus and stomach may decrease inappropriate LES relaxation by increasing the thickness of the muscular layer [17]. Endoscopic plication, such as EsophyX^®^, is a transoral incisionless fundoplication method performed using an endoscopic device. It reconstructs the LES in an attempt to restore the angle of His [17]. EsophyX^®^ may be effective for symptomatic GERD patients with Hill grades I–II or hiatal hernia < 2 cm [18]. The representative method among the techniques used for LES reinforcement is the LINX^®^ procedure, which uses magnetic attraction from inside a series of titanium beads to augment the weak LES and re-establish the natural barrier to reflux [19].

Endoscopic or surgical treatments for GERD are evidence-based procedures with proven efficacy in many RCTs [15]. However, there are few head-to-head trials on this topic, and the comparative efficacy of endoscopic and surgical treatments for GERD has not been fully evaluated. Therefore, we performed a network meta-analysis of RCTs of endoscopic and surgical treatments in patients with GERD, which can help rank these treatments according to their efficacy.

## 2. Methods

### 2.1. Study Design

A systematic review and network meta-analysis were conducted according to the Preferred Reporting Items for Systematic Reviews and Meta-Analyses statement [20] and the report of the International Society for Pharmacoeconomics and Outcomes Research Task Force on Indirect Treatment Comparisons Good Research Practices [21].

### 2.2. Search Strategy

All relevant studies published between January 1990 and August 2021 that evaluated the efficacy of endoscopic and surgical treatments for GERD were searched using the MEDLINE, EMBASE, and Cochrane Library databases. The following search string was used: ((reflux) OR (regurgitation) OR (GERD) OR (GORD)) AND ((radiofrequency) OR (stretta) OR (esophyX) OR ((transoral) AND ((plication) OR (fundoplication))) OR (endoscopic plication) OR (endoscopic fundoplication) OR (endoscopic gastroplication) OR (endoscopic full-thickness plication) OR (endoscopic full-thickness fundoplication) OR (plicator) OR (EndoCinch) OR (TIF) OR ((magnetic) AND (augmentation)) OR (MSA) OR (LINX) OR (endoscopic polymer implantation) OR (nonresorbable copolymer implantation) OR (esophageal prosthesis) OR (oesophageal prosthesis) OR (Enteryx) OR (Gatekeeper) OR (((surgical) OR (laparoscopic) OR (Nissen) OR (Toupet)) AND (fundoplication)) OR (total fundoplication) OR (partial fundoplication) OR (antireflux surgery) OR (anti-reflux surgery)) AND (random*). Appendix A presents the detailed search strategies for each database. The last date of updating our search was 14 August 2021.

### 2.3. Inclusion and Exclusion Criteria

The inclusion criteria were as follows: (a) population: patients with proven GERD; (b) intervention: endoscopic or surgical treatments including radiofrequency energy delivery, endoscopic plication, reinforcement of the LES, and surgical fundoplication; (c) comparator: PPI therapy or another type of endoscopic or surgical treatment; (d) outcome: requirement of PPI continuation, subjective outcomes (the GERD-health-related quality-of-life questionnaire (GERD-HRQL) score, a 36-item short-form survey (SF-36) physical component summary, and heartburn and regurgitation scores), and objective outcomes (esophageal erosion, abnormal acid exposure, and LES resting pressure); (e) study design: RCT, and (f) assessment timing: 3–12 months. The exclusion criteria were as follows: (a) studies involving only patients with Barrett’s esophagus; (b) studies that included non-proven GERD (for example, reflux hypersensitivity, functional heartburn); (c) non-original studies; (d) non-human studies; (e) abstract-only publications, and (f) non-English publications.

### 2.4. Study Selection

In the first step of study selection, duplicate articles retrieved through multiple search engines were excluded. Next, titles and abstracts of the articles were examined to exclude irrelevant studies. The full text of the remaining articles was reviewed for eligibility. Two investigators (E.J.G. and C.H.P.) independently evaluated the studies for eligibility and resolved any disagreements by discussion and consensus. If an agreement could not be reached, a third investigator (D.H.K.) determined the study eligibility. The Cochrane Risk of Bias assessment tool was used to assess the risk of bias in the included RCTs.

### 2.5. Data Extraction and Study Endpoint

Using a data extraction form developed in advance, two investigators (E.J.G. and C.H.P.) independently extracted the following information: first author, year of publication, study design, country, study period, publication language, types of intervention and comparator, assessment timing, and outcomes, including the requirement of PPI continuation and other clinical outcomes. Endoscopic or surgical treatments for GERD in individual studies were classified into the following four groups: (a) radiofrequency energy delivery (Stretta^®^), (b) endoscopic plication (EndoCinch^®^, NDO plicator^®^, EsophyX^®^, or GERD-X^®^), (c) reinforcement of LES (Enteryx^®^, Gatekeeper^®^, or LINX^®^), and (d) surgical fundoplication.

The primary endpoint in this meta-analysis was the comparative efficacy of endoscopic and surgical treatment in terms of the requirement of PPI continuation. The secondary endpoints were other clinical outcomes (GERD-HRQL score, SF36 physical component summary, heartburn and regurgitation scores, esophageal erosion, abnormal acid exposure, and LES resting pressure) and adverse events.

### 2.6. Statistical Analysis

A direct pairwise meta-analysis was conducted to calculate the pooled risk ratios (RRs) for categorical variables and the mean difference (MD) or standardized MD (SMD) for continuous variables, using a random-effects model. Statistical heterogeneity was assessed using the following two methods: Cochran’s Q test, in which *p*-values < 0.1 were considered statistically significant for heterogeneity, and *I*^2^ statistics, wherein values >50% suggested significant heterogeneity [22]. When the number of included studies for each pairwise comparison was <10, test for publication bias was not performed [23]. A direct pairwise meta-analysis was performed using Review Manager statistical software (version 5.3.5; Cochrane Collaboration, Copenhagen, Denmark).

A frequentist network meta-analysis was performed to calculate the direct and indirect estimates and combine the mixed estimates [24]. In addition, each treatment for GERD was ranked according to the P-scores, which were based solely on the point estimates and standard errors of the network estimates [25]. The P-score of each treatment can be interpreted as the mean extent of certainty that a certain treatment was better than another [25]. The network meta-analysis was conducted using the R statistical software (version 4.0.4; R Foundation for Statistical Computing, Vienna, Austria) with the netmeta package (version 2.0-1; Rücker et al.). The netmeta package is based on the graph theory methodology to model the relative treatment effects of multiple treatments under a frequentist framework [26].

## 3. Results

### 3.1. Study Selection and Characteristics

**Figure 1:**
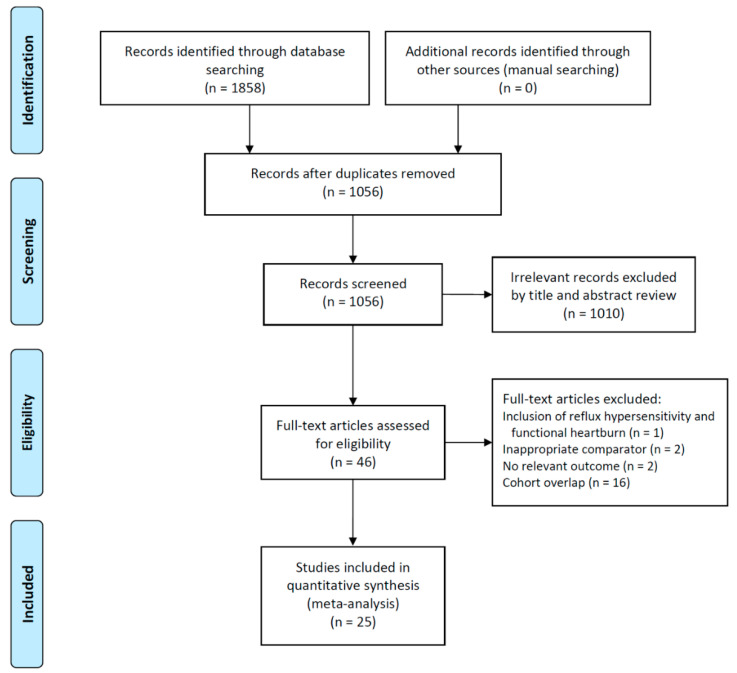
Flow diagram of the studies included in the meta-analysis.

**Figure 2:**
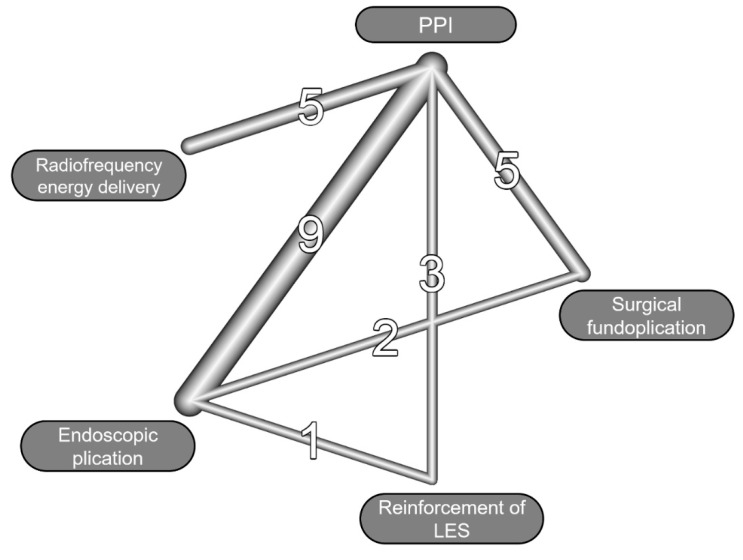
Evidence network of different treatments of gastroesophageal reflux disease. The line represents the comparison between different treatments. The thickness of the line and the numbers represent the number of studies included in each comparison. PPI, proton pump inhibitor; LES, lower esophageal sphincter.

Included in the meta-analysis were 25 studies, involving a total of 2854 patients (Figure 1) [27,28,29,30,31,32,33,34,35,36,37,38,39,40,41,42,43,44,45,46,47,48,49,50,51]; the baseline characteristics of the included studies are shown in Table 1. The studies were published between 2000 and 2021, with an enrollment period ranging from 1991 to 2019. Five studies compared radiofrequency energy delivery and PPI therapy [29,30,31], nine compared endoscopic plication and PPI therapy [32,33,34,35,36,37,38,39,40], three compared reinforcement of the LES and PPI therapy [41,42,43], and five discussed surgical fundoplication and PPI therapy [44,45,46,47,48]. One study evaluated the efficacy of endoscopic plication and reinforcement of the LES [49], whereas two studies evaluated the efficacy of endoscopic plication and surgical fundoplication [50,51]. Figure 2 illustrates the evidence network.

The risk of bias assessment for individual studies is shown in Figure S1. Among the 25 included studies, 1 (4%) had a high risk of bias in the domain of random sequence generation, because alternative allocation was performed. The other nine studies (36%) had an unclear risk of bias for random sequence generation. The risk of allocation concealment was unclear in seven studies (28%). Thirteen studies (52%) had a high risk of bias related to the blinding of participants because no sham controls were included in those studies.

Conversely, all studies were assessed as having a low risk of detection bias because the current study outcomes were less likely to be affected by the blinding of the investigators. All studies, except one (96%), showed that more than 75% of participants completed follow-up and were assessed as having a low risk of attrition bias. Reporting bias was not observed. Two studies (8%) were assessed as having a high risk of other biases because of early study termination.

### 3.2. Direct Meta-Analysis for the Efficacy of Endoscopic or Surgical Treatments

**Figure 3:**
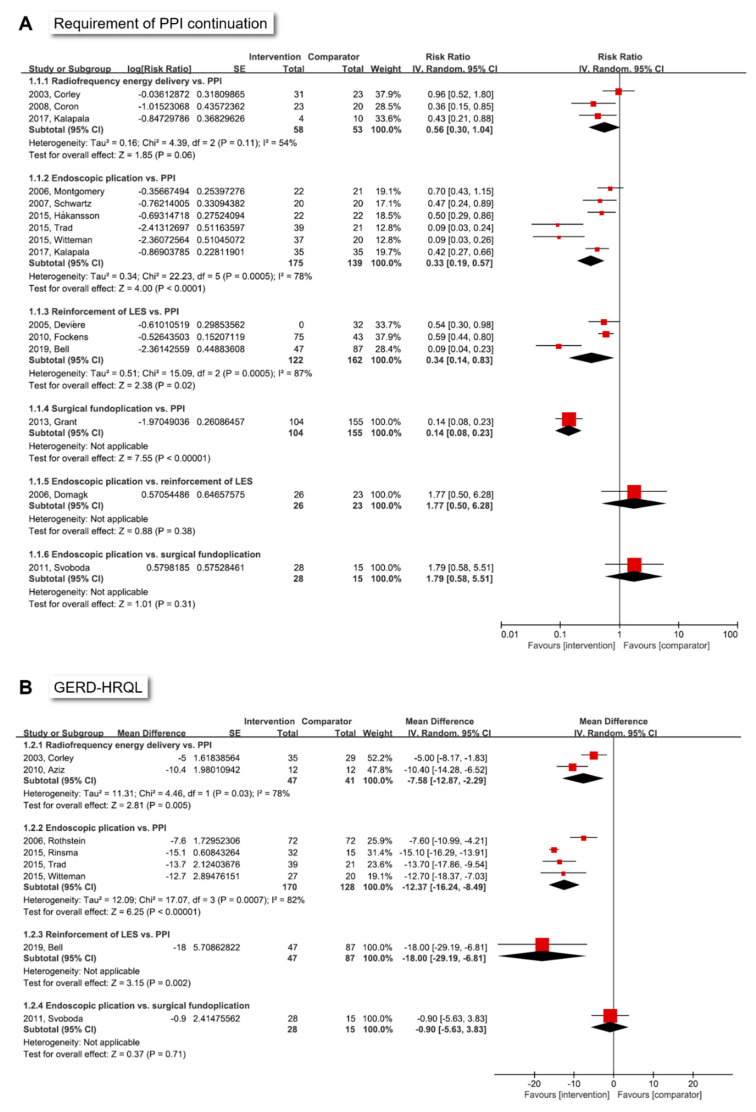
Direct meta-analysis of the requirement of PPI continuation (**A**) and GERD-HRQL (**B**) of endoscopic or surgical treatments. PPI, proton pump inhibitor; GERD, gastroesophageal reflux disease; HRQL, health-related quality of life questionnaire; LES, lower esophageal sphincter; SE, standard error; IV, inverse variance; CI, confidence interval; df, degrees of freedom.

The clinical outcomes of endoscopic and surgical treatments for GERD in individual studies are summarized in Table S1. In the direct meta-analysis, radiofrequency energy delivery tended to have a better efficacy regarding the requirement of PPI continuation compared to PPI therapy (pooled RR (95% confidence interval (CI)): 0.56 (0.30 to 1.04)) (Figure 3A). Endoscopic plication and reinforcement of the LES showed significantly better efficacy in terms of requirement of PPI continuation compared to PPI therapy (pooled RR (95% CI): endoscopic plication, 0.33 (0.19–0.57); reinforcement of the LES, 0.34 (0.14–0.83)). Heterogeneity across individual studies was observed in all three comparisons. Although the requirement of PPI continuation between surgical fundoplication and PPI therapy was assessed in only one study, surgical fundoplication appeared to be better than PPI therapy (RR (95% CI): 0.14 (0.08–0.23)). There was no difference in the requirement of PPI continuation between endoscopic plication and reinforcement of the LES, and between endoscopic plication and surgical fundoplication.

Direct meta-analysis results of the GERD-HRQL score, which indicate that the lower the value, the better the quality of life, are shown in Figure 3B. Both radiofrequency energy delivery and endoscopic plication were superior in terms of GERD-HRQL compared to PPI therapy (pooled MD (95% CI): radiofrequency energy delivery, −7.58 (−12.87 to −2.29); and endoscopic plication, −12.37 (−16.24 to −8.49)). Although there was only one study evaluating GERD-HRQL between reinforcement of the LES and PPI therapy, LES reinforcement appeared to be superior in terms of GERD-HRQL compared to PPI therapy (MD (95% CI): −18.00 (−29.19 to −6.81)). No study has compared surgical fundoplication and PPI therapy.

Other subjective outcomes, including the SF-36 physical component summary, heartburn score, and regurgitation score, are shown in Figure S2. Although a limited number of studies were included in most comparisons, radiofrequency energy delivery, endoscopic plication, reinforcement of the LES, and surgical fundoplication had significant effects or tended to be effective in controlling subjective symptoms, including heartburn and regurgitation. Figure S3 shows the direct meta-analysis of objective outcomes, including esophageal erosion, abnormal acid exposure, and LES resting pressure. Surgical fundoplication reduced abnormal acid exposure and increased LES resting pressure compared to PPI therapy, whereas endoscopic treatments failed to show better objective outcomes compared to PPI therapy.

### 3.3. Network Meta-Analysis for the Efficacy of Endoscopic or Surgical Treatments

**Figure 4:**
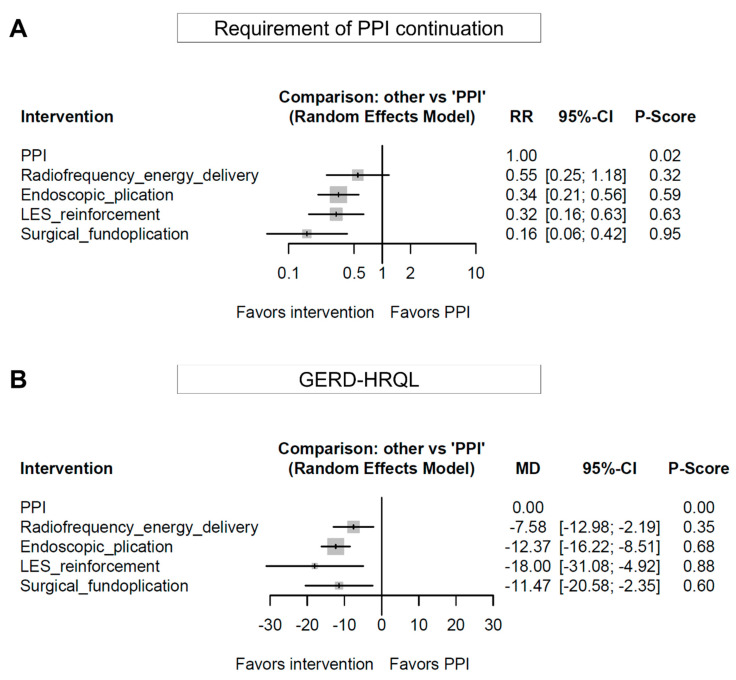
Comparative efficacy for the requirement of PPI continuation (**A**) and GERD-HRQL score (**B**) in the network meta-analysis. The P-score indicates the mean extent of certainty that one treatment is better than another. PPI, proton pump inhibitor; GERD, gastroesophageal reflux disease; HRQL, health-related quality of life questionnaire; LES, lower esophageal sphincter; RR, risk ratio; MD, mean difference; CI, confidence interval.

Table S2 shows the network estimates for the clinical outcomes of endoscopic or surgical treatments for GERD. Forest plots of the network meta-analysis for the requirement of PPI continuation and GERD-HRQL are shown in Figure 4. Compared to PPI therapy, radiofrequency energy delivery tended to reduce the requirement of PPI continuation (pooled RR (95% CI): 0.55 (0.25–1.18)). Endoscopic plication, reinforcement of LES, and surgical fundoplication were effective in reducing the requirement for PPI continuation compared to PPI therapy (pooled RR (95% CI): endoscopic plication, 0.34 (0.21–0.56); reinforcement of the LES, 0.32 (0.16–0.63), and surgical fundoplication, 0.16 (0.06–0.42)). The P-score was the highest for surgical fundoplication (95%), followed by LES reinforcement (63%), endoscopic plication (59%), radiofrequency energy delivery (32%), and PPI therapy (2%). Significant network inconsistency was identified in the network meta-analysis (*p* < 0.001, *I*^2^ = 74.8%). With regard to the GERD-HRQL score, all endoscopic and surgical treatments showed better efficacy than PPI therapy (pooled MD (95% CI): radiofrequency energy delivery, −7.58 (−12.98 to −2.19); endoscopic plication, −12.37 (−16.22 to −8.51); LES reinforcement, −18.00 (−31.08 to −4.92), and surgical fundoplication, −11.47 (−20.58 to −2.35)). Network inconsistency was also identified in the network meta-analysis (*p* < 0.001, *I*^2^ = 81.4%).

Forest plots of network meta-analysis for other subjective outcomes, including the SF-36 physical component summary, heartburn score, and regurgitation score, are shown in Figure S4. Surgical fundoplication showed a better SF-36 physical component summary compared to PPI therapy (MD (95% CI): 2.81 (0.64–4.99)). Other treatments, including radiofrequency energy delivery, endoscopic plication, and reinforcement of the LES, showed a tendency of better SF-36 physical component summary compared to PPI therapy. However, these differences were not statistically significant. For heartburn score, radiofrequency energy delivery and surgical fundoplication were superior to PPI therapy (SMD (95% CI): radiofrequency energy delivery, −1.26 (−2.20 to −0.32); surgical fundoplication, −1.37 (−2.47 to −0.26)). In the meta-analysis of objective outcomes, including esophageal erosion, abnormal acid exposure, and LES resting pressure, endoscopic treatments did not show better efficacy than PPI therapy (Figure S5). In contrast, surgical fundoplication showed less abnormal acid exposure and higher LES resting pressure than did PPI therapy.

### 3.4. Sensitivity Analysis

Two sensitivity analyses were performed for primary outcomes. Figure S6A shows the comparative efficacy of PPI continuation, after excluding seven non-participant-blinding studies. Even in this sensitivity analysis, endoscopic plication and reinforcement of the LES showed less requirement for PPI continuation than PPI therapy. Radiofrequency energy delivery tended to have less requirement for PPI continuation than PPI therapy, although the difference was not statistically significant. In an additional sensitivity analysis, after two early terminated studies were excluded, continuation of PPI was less required in participants who underwent endoscopic plication, reinforcement of the LES, or surgical fundoplication, compared to those who only received PPI therapy (Figure S6B). In this sensitivity analysis, patients who underwent radiofrequency energy delivery also tended to have a lower requirement for PPI continuation compared to PPI therapy.

### 3.5. Adverse Events

Adverse events associated with endoscopic or surgical treatments for GERD are summarized in Table S3. The most common major adverse event after endoscopic treatment was transient chest or abdominal pain. Although one patient died 11 months after receiving endoscopic plication, the exact cause remains unclear.

## 4. Discussion

In this study, various treatment outcomes were evaluated, including the requirement of PPI continuation and GERD-HRQL, in patients with GERD who underwent endoscopic or surgical treatment. In terms of the requirement for PPI continuation, surgical fundoplication, LES reinforcement, and endoscopic plication were better than PPI therapy. These results imply that surgical fundoplication, LES reinforcement, or endoscopic plication may be performed to avoid PPI therapy in patients with GERD. Although not statistically significant, radiofrequency energy delivery may also be an alternative option. According to the P-scores, surgical fundoplication was the best option for PPI discontinuation (95%), followed by LES reinforcement (63%), endoscopic plication (59%), and radiofrequency energy delivery (32%). However, the rank of endoscopic or surgical treatments should be interpreted with caution because there were no significant differences among these treatments. For example, PPI continuation tended to be less required in patients who underwent surgical fundoplication than in those who underwent reinforcement of the LES, endoscopic plication, or radiofrequency energy delivery; however, these differences were not statistically significant. Additionally, endoscopic treatments are usually less invasive than surgical fundoplication. Clinicians may recommend endoscopic or surgical treatment for PPI-dependent patients considering their performance status and preference. In other words, a tailored approach to patients with PPI-dependent or refractory GERD may be beneficial for improving treatment outcomes.

Endoscopic or surgical treatments for GERD also have better efficacy in terms of GERD-HRQL than PPI therapy. This finding is relevant for clinicians and GERD patients because quality of life usually deteriorates in patients with GERD, despite receiving acid-suppressive medications [11]. If patients with GERD are not satisfied with PPI therapy, endoscopic or surgical treatment options should be considered. In addition to GERD-HRQL, other subjective outcomes, including the SF-36 physical component summary and heartburn score, were better in patients who underwent endoscopic or surgical treatments than in those who received PPI therapy, although statistical significance was not achieved in some of these comparisons.

In contrast to the results mentioned above, radiofrequency energy delivery, endoscopic plication, and LES reinforcement did not show better efficacy in terms of objective outcomes, including abnormal acid exposure and resting pressure of the LES. Only surgical fundoplication showed a higher resting pressure of the LES and less abnormal acid exposure than PPI therapy. These results imply that treatments other than surgical fundoplication may be insufficient to prevent pathological acid reflux. In clinical practice, however, normalization of abnormal acid exposure is not an endpoint in the management of GERD [16]. Given that symptom relief and discontinuation of PPIs are the ultimate goals of GERD treatment, both endoscopic and surgical treatments may be considered in PPI-dependent or PPI-refractory patients with GERD. Meanwhile, esophageal erosion did not differ between endoscopic or surgical treatments and PPI therapy. This finding may be attributable to the fact that erosive esophagitis is controlled well, even with PPI therapy alone.

Although several beneficial efficacy outcomes of endoscopic or surgical treatments for GERD have been shown in this network meta-analysis, our results do not indicate that PPI therapy is a poor treatment option for patients with GERD. In the LOTUS trial, which compared surgical fundoplication with PPI therapy, the symptom remission rate remained high in both groups after 5 years of treatment [14]. Although medical treatment with PPIs is unlikely to discontinue the requirement for medication compared to endoscopic or surgical treatments, GERD symptoms can be well controlled by PPI therapy alone. Additionally, we do not recommend endoscopic or surgical treatments for all patients with GERD because most of the included studies described patients with PPI-dependent or PPI-refractory GERD. If previous RCTs were conducted on patients with mild GERD who responded well to PPI therapy, the requirement of PPI continuation or subjective outcomes, including GERD-HRQL, may not differ between endoscopic or surgical treatments and PPI therapy. Therefore, only patients with PPI-dependent or PPI-refractory GERD should be candidates for endoscopic or surgical treatments [6]. Additionally, potential adverse events and the medical cost of endoscopic or surgical treatments can be a hurdle in performing these procedures for GERD [52]; however, most adverse events reported in the previous RCTs were not serious. In addition, endoscopic or surgical treatments may be cost effective in PPI-dependent patients who require long-term medication. Previous studies have shown that surgical fundoplication is likely to be an overall cost-effective option, despite its high initial cost [7,47].

Although this is the first network meta-analysis to cover various endoscopic or surgical treatments for GERD, it has several limitations. First, although many RCTs have investigated endoscopic or surgical treatments for GERD, only a few head-to-head trials comparing these treatments have been included. Therefore, the efficacy for some of the comparisons (e.g., endoscopic plication vs. radiofrequency energy delivery) was derived from indirect estimates using a common comparator (PPI therapy). Therefore, further head-to-head trials are required to reach a definitive conclusion. Second, the reported outcomes varied across studies, and a relatively small number of studies were included in the comparisons. Third, the long-term follow-up data of patients undergoing endoscopic treatments were insufficient. Most studies on endoscopic treatments have assessed outcomes 3–12 months after treatment. Therefore, studies on surgical fundoplication that reported long-term (>12 months) outcomes were not included, in order to ensure comparability between studies. The long-term comparative efficacy between endoscopic and surgical treatments will be estimated when follow-up data for endoscopic treatments are accumulated. Fourth, several procedures, such as NDO plicator^®^, Enteryx^®^, and Gatekeeper^®^, are no longer commercially available owing to the lack of long-term data, adverse events, or the company’s poor financial performance [17]. However, through this network meta-analysis, we can understand the efficacy of currently available procedures, including Stretta^®^ and EsophyX^®^, compared to other techniques.

Despite these limitations, this network meta-analysis provides a better understanding of the efficacy of various endoscopic and surgical treatments in patients with PPI-dependent or PPI-refractory GERD. All endoscopic and surgical treatments, except for radiofrequency energy delivery, were effective for discontinuation of PPI medication, especially surgical fundoplication. Quality of life, measured by GERD-HRQL, also improved in patients who underwent endoscopic or surgical treatments compared to those who received PPI therapy. Endoscopic or surgical treatment may be considered in patients with PPI-dependent or PPI-refractory GERD, as an approach to discontinue PPI therapy and improve these patients’ quality of life.

## Figures and Tables

**Table 1:** Baseline characteristics of the included studies.

Publication Year, First Author [Reference Number]	Country	Study Period	Study Population	Number of Participants	Arm 1	Arm 2	Age, Year	Male, %
2003, Corley [27]	USA	2000–2001	PPI-dependent patients with GERD	64	Radiofrequency energy delivery	Sham + PPI	Arm 1: mean 45 (SD 12) Arm 2: mean 52 (SD 15)	51.6
2008, Coron [28]	France	2003–2006	PPI-dependent patients with GERD	43	Radiofrequency energy delivery	PPI	Arm 1: mean 50 (SD 10) Arm 2: mean 47 (SD 14)	69.8
2010, Aziz [29]	USA	2005–2006	PPI-dependent patients with GERD	24	Radiofrequency energy delivery	Sham + PPI	Arm 1: mean 36.7 (SD 9.5) Arm 2: mean 32.0 (SD 8.3)	66.7
2012, Arts [30]	Belgium	N/A	Patients with GERD	22	Radiofrequency energy delivery	Sham + PPI	Mean 46.5 (SD 2.4)	22.7
2017, Kalapala [31]	India	2015–2016	Patients with refractory GERD	20	Radiofrequency energy delivery	Sham + PPI	Mean 36.5 (SD 13.0)	100.0
2006, Montgomery [32]	Sweden	N/A	PPI-dependent patients with GERD	46	Endoscopic plication	Sham + PPI	Arm 1: median 42 (range 22–66) Arm 2: median 41 (range 19–66)	32.6
2006, Rothstein [33]	USA and Europe	2005	PPI-dependent patients with GERD	144	Endoscopic plication	Sham + PPI	Arm 1: mean 48.1 (SD 13.1) Arm 2: mean 46.3 (SD 13.8)	52.1
2007, Schwartz [34]	The Netherlands	2003–2005	PPI-dependent patients with GERD	60	Endoscopic plication	Sham + PPI	Arm 1: mean 45 (SD 12) Arm 2: mean 47 (SD 12)	62.5
2015, Håkansson [35]	Sweden	2011–2013	PPI-dependent patients with GERD	44	Endoscopic plication	Sham + PPI	Arm 1: median 41 (range 21–67) Arm 2: median 62 (range 31–76)	54.5
2015, Hunter [36]	USA	2011–2013	Patients with refractory GERD	129	Endoscopic plication	Sham + PPI	Arm 1: median 52 (range 22–74) Arm 2: median 55 (range 22–73)	48.8
2015, Rinsma [37]	The Netherlands	2008–2012	PPI-dependent patients with GERD	47	Endoscopic plication	PPI	Mean 45 (range 19–68)	63.8
2015, Trad [38]	USA	2012	Patients with refractory GERD	60	Endoscopic plication	PPI	Arm 1: median 54.8 (range 35.7–73.3) Arm 2: median 50.1 (range 32.5–63.3)	45.0
2015, Witteman [39]	The Netherlands and USA	2008–2011	PPI-dependent patients with GERD	60	Endoscopic plication	PPI	Mean 44.7 (SD 12.9)	63.3
2021, Kalapala [40]	India	2017–2019	PPI-dependent patients with GERD	70	Endoscopic plication	Sham + PPI	Median 36 (IQR 29–42)	71.4
2005, Devière [41]	Germany, Beligum, and Italy	2001–2004	PPI-dependent patients with GERD	64	Reinforcement of the LES	Sham + PPI	Arm 1: mean 49.7 (SD 14.2) Arm 2: mean 48.6 (SD 10.2)	67.2
2010, Fockens [42]	USA and the Netherlands	2003–2005	PPI-dependent patients with GERD	118	Reinforcement of the LES	Sham + PPI	Arm 1: mean 47.9 (SD 11.6) Arm 2: mean 52.6 (SD 11.8)	63.6
2019, Bell [43]	USA	2015–2017	Patients with refractory GERD	152	Reinforcement of the LES	PPI	Median 46 (range 21–76)	56.6
2000, Lundell [44]	Europe	1991–1999	Patients with GERD	298	Surgical fundoplication	PPI	N/A	75.5
2005, Mahon [45]	UK	1997–2001	PPI-dependent patients with GERD	217	Surgical fundoplication	PPI	Arm 1: median 48 (IQR 39–56) Arm 2: median 47 (IQR 35–57)	69
2006, Anvari [46]	Canada	2000–2004	PPI-dependent patients with GERD	104	Surgical fundoplication	PPI	Arm 1: mean 42.9 Arm 2: mean 42.1	52.9
2013, Grant [47]	UK	2001–2004	PPI-dependent patients with GERD	357	Surgical fundoplication	PPI	Arm 1: mean 46.7 (SD 10.3) Arm 2: mean 45.9 (SD 11.9)	66.1
2016, Hatlebakk [48]	Europe	2001–2009	Patients with GERD	554	Surgical fundoplication	PPI	Mean 45.1 (SD 11.2)	71.8
2006, Domagk [49]	Germany	2002–2005	PPI-dependent patients with GERD	49	Endoscopic plication	Reinforcement of the LES	Mean 48 (SD 15)	53.1
2011, Svoboda [50]	Czech	2007–2009	Patients with GERD	52	Endoscopic plication	Surgical fundoplication	Arm 1: median 49 (range 25–69) Arm 2: median 55 (range 39–70)	48.1
2012, Antoniou [51]	Austria	2006–2010	Patients with GERD	56	Endoscopic plication	Surgical fundoplication	Arm 1: mean 46.5 Arm 2: mean 46.3	N/A

PPI, proton pump inhibitor; LES, lower esophageal sphincter; SD, standard deviation; IQR, interquartile range; N/A, not available.

## Data Availability

All relevant data are included in the study and supplementary information.

## Supplementary Materials

The following supporting information can be downloaded at: https://www.mdpi.com/article/10.3390/jpm12040621/s1, Figure S1: Risk of bias summary; Figure S2: Direct meta-analysis of subjective outcomes of endoscopic or surgical treatments; Figure S3: Direct meta-analysis of objective outcomes of endoscopic or surgical treatments; Figure S4: Comparative efficacy for subjective outcomes in the network meta-analysis; Figure S5: Comparative efficacy for objective outcomes in the network meta-analysis; Figure S6: Sensitivity analyses of the requirement of PPI continuation; Table S1: Clinical outcomes of endoscopic or surgical treatments in the included studies; Table S2: Network estimates of the endoscopic or surgical treatments of GERD; Table S3: Adverse events of endoscopic or surgical treatments of GERD.

## Appendix A. Detailed Search Strategy

MEDLINE (Search interface: PubMed)
(reflux[tw] OR regurgitation[tw] OR GERD[tw] OR GORD[tw]) AND (radiofrequency[tw] OR stretta[tw] OR esophyX[tw] OR (transoral[tw] AND (plication[tw] OR fundoplication[tw])) OR (endoscopic plication[tw]) OR (endoscopic fundoplication[tw]) OR (endoscopic gastroplication[tw]) OR (endoscopic full-thickness plication[tw]) OR (endoscopic full-thickness fundoplication[tw]) OR plicator[tw] OR EndoCinch[tw] OR TIF[tw] OR (magnetic[tw] AND augmentation[tw]) OR MSA[tw] OR LINX[tw] OR (endoscopic polymer implantation[tw]) OR (nonresorbable copolymer implantation[tw]) OR (esophageal prosthesis[tw]) OR (oesophageal prosthesis[tw]) OR Enteryx[tw] OR Gatekeeper[tw] OR ((surgical[tw] OR laparoscopic[tw] OR Nissen[tw] OR Toupet[tw]) AND fundoplication[tw]) OR (total fundoplication[tw]) OR (partial fundoplication[tw]) OR (antireflux surgery[tw]) OR (anti-reflux surgery[tw])) AND random*[tw] AND (“1990/01/01”[Date - Publication] : “3000”[Date - Publication])
EMBASE (Search interface: Ovid)
1: ((reflux or regurgitation or GERD or GORD) and (radiofrequency or stretta or esophyX or (transoral and (plication or fundoplication)) or endoscopic plication or endoscopic fundoplication or endoscopic gastroplication or endoscopic full-thickness plication or endoscopic full-thickness fundoplication or plicator or EndoCinch or TIF or (magnetic and augmentation) or MSA or LINX or endoscopic polymer implantation or nonresorbable copolymer implantation or esophageal prosthesis or oesophageal prosthesis or Enteryx or Gatekeeper or ((surgical or laparoscopic or Nissen or Toupet) and fundoplication) or total fundoplication or partial fundoplication or antireflux surgery or anti-reflux surgery) and random*).ab,ti.
2: Limit 1 to (english language and embase and yr=“1990 -Current”)
Cochrane library
#1: reflux or regurgitation or GERD or GORD
#2: radiofrequency or stretta or esophyX
#3: transoral and plication
#4: transoral and fundoplication
#5: #3 or #4
#6: ‘endoscopic plication’ or ‘endoscopic fundoplication’ or ‘endoscopic gastroplication’ or ‘endoscopic full-thickness plication’ or ‘endoscopic full-thickness fundoplication’ or plicator or EndoCinch or TIF
#7: magnetic and augmentation
#8: MSA or LINX
#9: ‘endoscopic polymer implantation’ or ‘nonresorbable copolymer implantation’ or ‘esophageal prosthesis’ or ‘oesophageal prosthesis’ or Enteryx or Gatekeeper
#10: surgical or laparoscopic or Nissen or Toupet
#11: fundoplication
#12: #10 and #11
#13: ‘total fundoplication’ or ‘partial fundoplication’ or ‘antireflux surgery’ or ‘anti-reflux surgery’
#14: #2 or #5 or #6 or #7 or #8 or #9 or #12 or #13
#15: random*
#16: #1 and #14 and #15 (with Publication Year from 1990 to 2021, in Trials)
