# Supplementary material for: Efficacy of Endoscopic and Surgical Treatments for Gastroesophageal Reflux Disease: A Systematic Review and Network Meta-Analysis
Source: J Pers Med. 2022 Apr 12;12(4):621. doi: 10.3390/jpm12040621 (PMC9031147; doi:10.3390/jpm12040621)
Supplement: Supplementary file 1 [file jpm-12-00621-s001.zip › 04. GERD intervention - Table S1.pdf]

Table S1. Clinical outcomes of endoscopic or surgical treatments in the included studies

Publication year, First author [reference number]	Arm 1	Arm 2	Assessment timing	Requirement of PPI continuation (event / N)		GERD-HRQL		SF36 physical component summary		Heartburn score			Regurgitation score			Esophageal erosion (event / N)		Abnormal acid exposure (event / N)		LES resting pressure, mmHg	
				Arm 1	Arm 2	Arm 1	Arm 2	Arm 1	Arm 2	Measure	Arm 1	Arm 2	Measure	Arm 1	Arm 2	Arm 1	Arm 2	Arm 1	Arm 2	Arm 1	Arm 2
2003, Corley [23]	Radiofrequency energy delivery	Sham + PPI	6 months	13 / 31	10 / 23	Mean 16 (95% CI: 12, 20)	Mean 21 (95% CI: 16, 26)	Mean 47 (95% CI: 43, 51)	Mean 42 (95% CI: 36, 47)	6-point Likert scale	Mean 2.2 (95% CI: 1.6, 2.7)	Mean 2.8 (95% CI: 2.2, 3.5)				5 / 25	5 / 23			Median 16.2 (IQR, 10.6-23.0)	Median 18.0 (IQR, 14.8-22.5)
2008, Coron [24]	Radiofrequency energy delivery	PPI	6 months	5 / 23	12 / 20			Mean 48 (SD 8)	Mean 49 (SD 7)	5-point Likert scale	Mean 2.1 (SD 1.0)	Mean 2.4 (SD 1.4)	5-point Likert scale	Mean 1.3 (SD 0.6)	Mean 2.2 (SD 1.3)	10 / 19	7 / 13	17 / 18	9 / 12		
2010, Aziz [25]	Radiofrequency energy delivery	Sham + PPI	12 months			Mean 14.4 (SD 4.8)	Mean 24.8 (SD 4.9)									8 / 12	9 / 12	7 / 12	12 / 12	Mean 16.2 (SD 4.5)	Mean 15.9 (SD 3.2)
2012, Arts [26]	Radiofrequency energy delivery	Sham + PPI	3 months													4 / 11	4 / 11	11 / 11	11 / 11	Mean 13.3 (SD 1.9)	Mean 16.3 (SD 2.0)
2017, Kalapala [27]	Radiofrequency energy delivery	Sham + PPI	3 months	4 / 10	10 / 10					6-point Likert scale	Mean 0.60 (SD 0.84)	Mean 3.80 (SD 0.63)	6-point Likert scale	Mean 0.50 (SD 0.71)	Mean 3.30 (SD 0.68)					Mean 9.1 (SD 5.75)	Mean 9.44 (SD 3.14)
2006, Montgomery [28]	Endoscopic plication	Sham + PPI	3 months	11 / 22	15 / 21											6 / 22	2 / 24			Median 11.2 (IQR 7.5-15.9)	Median 13.9 (IQR 9.7-15.8)
2006, Rothstein [29]	Endoscopic plication	Sham + PPI	3 months			Mean 12.5 (SD 11.1)	Mean 20.1 (SD 9.6)	Mean 49.9 (SD 10.5)	Mean 46.1 (SD 9.1)	Visual analogue scale ranged 0-100	35.5 (SD 27.0)	58.3 (SD 24.4)									
2007, Schwartz [30]	Endoscopic plication	Sham + PPI	3 months	7 / 20	15 / 20													12 / 17	14 / 18		
2015, Håkansson [31]	Endoscopic plication	Sham + PPI	6 months	9 /22	18 / 22													5 / 15	9 /11		
2015, Hunter [32]	Endoscopic plication	Sham + PPI	6 months													7 / 17	6 / 7				
2015, Rinsma [33]	Endoscopic plication	PPI	6 months			Mean 8.5 (SD 1.3)	Mean 23.6 (SD 2.9)														

2015, Trad [34]	Endoscopic plication	PPI	6 months	3 / 39	21 / 21	Mean 5.2 (SD 7.1)	Mean 18.9 (SD 9.1)			GERD-HRQL heartburn domain	Mean 3.7 (SD 5.5)	Mean 11.7 (SD 6.94)						18 / 39	10 / 21		
2015, Witteman [35]	Endoscopic plication	PPI	6 months	3 / 37	20 / 20	Mean 12.4 (SD 10.0)	Mean 25.1 (SD 11.2)									5 / 37	2 / 20	18 / 37	7 / 20	Median 18.2 (range 10-40)	Median 13.6 (range 4-20)
2021, Kalapala [36]	Endoscopic plication	Sham + PPI	12 months	13 / 35	31 / 35											0 / 18	5 / 17				
2005, Devière [37]	Reinforcement of LES	Sham + PPI	3 months	10 / 31	19 / 32																
2010, Fockens [38]	Reinforcement of LES	Sham + PPI	6 months	34 / 75	33 / 43			Mean 49.8 (SD 7.7)	Mean 49.4 (SD 9.0)	GERD-HRQL heartburn domain	Mean 13.7 (SD 10.1)	Mean 16.4 (SD 9.7)	GERD-HRQL regurgitation domain	Mean 8.8 (SD 7.5)	Mean 11.1 (SD 7.9)					Mean 17.0 (SD 10.9)	Mean 16.8 (SD 10.8)
2019, Bell [39]	Reinforcement of LES	PPI	6 months	4 / 47	87 / 87	Mean 6	Mean 24											5 / 44	20 / 79		
2000, Lundell [40]	Fundoplication	PPI	12 months													23 / 133	21 / 145				
2005, Mahon [41]	Laparoscopic Nissen fundoplication	PPI	3 months		100 / 108															Mean 17.2 (SD 7.0)	Mean 7.9 (SD 7.7)
2006, Anvari [42]	Laparoscopic Nissen fundoplication	PPI	12 months					Mean 46.4 (SD 10.9)	Mean 43.9 (SD 10.3)	GERSS heartburn domain	Mean 0.8 (SD 1.8)	Mean 2.8 (SD 2.6)	GERSS regurgitation domain	Mean 0.7 (SD 1.6)	Mean 2.7 (SD 2.8)					MD 8.97 (95% CI: 6.37, 11.57)	
2013, Grant [43]	Laparoscopic fundoplication	PPI	12 months	13 / 104	139 / 155			Mean 48.0 (SD 10.2)	Mean 45.1 (SD 9.7)												
2016, Hatlebakk [44]	Laparoscopic fundoplication	PPI	6 months		151 / 151													12 / 116	51 / 151		
2006, Domagk [45]	Endoscopic plication	Reinforcement of LES	6 months	6 / 26	3 / 23			Mean 50.3 (SD 8.1)	Mean 49.5 (SD 7.9)	Composite score ranged 0-30 (severity and frequency)	Mean 20.9 (SD 24.2)	Mean 19.9 (SD 24.4)								Mean 38.4 (SD 10.6)	Mean 25.4 (SD 10.1)
2011, Svoboda [46]	Endoscopic plication	Laparoscopic Nissen fundoplication	3 months	10 / 28	3 / 15	Mean 5.8 (SD 6.3)	Mean 6.7 (SD 9.5)														

2012, Antoniou [47]	Endoscopic plication	Laparoscopic fundoplication	3 months	5-point Likert scale	Mean 1.04	Mean 0.04	5-point Likert scale	Mean 0.56	Mean 0.08

PPI, proton pump inhibitor; LES, lower esophageal sphincter; GERD, gastroesophageal reflux disease; HRQL, health-related quality of life questionnaire; SF36, 36-item short-form survey; SD, standard deviation; IQR, interquartile range; N/A, not available
