# Supplementary material for: Efficacy of Endoscopic and Surgical Treatments for Gastroesophageal Reflux Disease: A Systematic Review and Network Meta-Analysis
Source: J Pers Med. 2022 Apr 12;12(4):621. doi: 10.3390/jpm12040621 (PMC9031147; doi:10.3390/jpm12040621)
Supplement: Supplementary file 1 [file jpm-12-00621-s001.zip › 04. GERD intervention - Table S2.pdf]

Table S2. Network estimates of the endoscopic or surgical treatments of GERD

Variable	Compared treatment				
	vs. PPI	vs. Radiofrequency energy delivery	vs. Endoscopic plication	vs. Reinforcement of LES	vs. Surgical fundoplication
Outcome: Requirement of PPI continuation, RR (95% CI)					
PPI	.	1.83 (0.84, 3.96)	2.94 (1.78, 4.83)	3.11 (1.58, 6.13)	6.38 (2.37, 17.18)
Radiofrequency energy delivery	0.55 (0.25, 1.18)	.	1.61 (0.64, 4.03)	1.70 (0.61, 4.76)	3.49 (0.99, 12.26)
Endoscopic plication	0.34 (0.21, 0.56)	0.62 (0.25, 1.56)	.	1.06 (0.48, 2.34)	2.17 (0.78, 6.04)
Reinforcement of LES	0.32 (0.16, 0.63)	0.59 (0.21, 1.64)	0.94 (0.43, 2.08)	.	2.05 (0.62, 6.72)
Surgical fundoplication	0.16 (0.06, 0.42)	0.29 (0.08, 1.01)	0.46 (0.17, 1.28)	0.49 (0.15, 1.60)	.
Outcome: GERD-HRQL, MD (95% CI)					
PPI	.	7.58 (2.19, 12.98)	12.37 (8.51, 16.22)	18.00 (4.92, 31.08)	11.47 (2.35, 20.58)
Radiofrequency energy delivery	-7.58 (-12.98, -2.19)	.	4.78 (-1.85, 11.42)	10.42 (-3.73, 24.56)	3.88 (-6.71, 14.48)
Endoscopic plication	-12.37 (-16.22, -8.51)	-4.78 (-11.42, 1.85)	.	5.63 (-8.00, 19.27)	-0.90 (-9.16, 7.36)
Reinforcement of LES	-18.00 (-31.08, -4.92)	-10.42 (-24.56, 3.73)	-5.63 (-19.27, 8.00)	.	-6.53 (-22.47, 9.41)
Surgical fundoplication	-11.47 (-20.58, -2.35)	-3.88 (-14.48, 6.71)	0.90 (-7.36, 9.16)	6.53 (-9.41, 22.47)	.
Outcome: SF36 physical component summary, MD (95% CI)					
PPI	.	-2.10 (-5.71, 1.50)	-2.88 (-5.84, 0.07)	-1.12 (-4.40, 2.15)	-2.81 (-4.99, -0.64)
Radiofrequency energy delivery	2.10 (-1.50, 5.71)	.	-0.78 (-5.45, 3.88)	0.98 (-3.89, 5.86)	-0.71 (-4.92, 3.50)
Endoscopic plication	2.88 (-0.07, 5.84)	0.78 (-3.88, 5.45)	.	1.76 (-1.70, 5.22)	0.07 (-3.60, 3.74)
Reinforcement of LES	1.12 (-2.15, 4.40)	-0.98 (-5.86, 3.89)	-1.76 (-5.22, 1.70)	.	-1.69 (-5.62, 2.24)
Surgical fundoplication	2.81 (0.64, 4.99)	0.71 (-3.50, 4.92)	-0.07 (-3.74, 3.60)	1.69 (-2.24, 5.62)	.
Outcome: Heartburn score, SMD (95% CI)					
PPI	.	1.26 (0.32, 2.20)	0.74 (-0.10, 1.57)	0.52 (-0.59, 1.62)	1.37 (0.26, 2.47)
Radiofrequency energy delivery	-1.26 (-2.20, -0.32)	.	-0.53 (-1.78, 0.73)	-0.74 (-2.19, 0.71)	0.11 (-1.34, 1.55)
Endoscopic plication	-0.74 (-1.57, 0.10)	0.53 (-0.73, 1.78)	.	-0.22 (-1.33, 0.89)	0.63 (-0.48, 1.74)
Reinforcement of LES	-0.52 (-1.62, 0.59)	0.74 (-0.71, 2.19)	0.22 (-0.89, 1.33)	.	0.85 (-0.60, 2.30)
Surgical fundoplication	-1.37 (-2.47, -0.26)	-0.11 (-1.55, 1.34)	-0.63 (-1.74, 0.48)	-0.85 (-2.30, 0.60)	.
Outcome: Regurgitation score, SMD (95% CI)					
PPI	.	2.40 (-0.62, 5.43)	0.10 (-5.76, 5.97)	0.30 (-3.84, 4.45)	0.89 (-3.26, 5.03)
Radiofrequency energy delivery	-2.40 (-5.43, 0.62)	.	-2.30 (-8.90, 4.30)	-2.10 (-7.24, 3.03)	-1.52 (-6.65, 3.61)
Endoscopic plication	-0.10 (-5.97, 5.76)	2.30 (-4.30, 8.90)	.	0.20 (-6.98, 7.38)	0.78 (-3.37, 4.94)
Reinforcement of LES	-0.30 (-4.45, 3.84)	2.10 (-3.03, 7.24)	-0.20 (-7.38, 6.98)	.	0.58 (-5.27, 6.44)
Surgical fundoplication	-0.89 (-5.03, 3.26)	1.52 (-3.61, 6.65)	-0.78 (-4.94, 3.37)	-0.58 (-6.44, 5.27)	.
Outcome: Esophageal erosion, RR (95% CI)					
PPI	.	1.07 (0.66, 1.74)	1.37 (0.70, 2.69)	Not estimated	0.84 (0.38, 1.86)
Radiofrequency energy delivery	0.94 (0.58, 1.52)	.	1.28 (0.56, 2.95)	Not estimated	0.78 (0.31, 2.00)
Endoscopic plication	0.73 (0.37, 1.43)	0.78 (0.34, 1.79)	.	Not estimated	0.61 (0.21, 1.74)
Reinforcement of LES	Not estimated	Not estimated	Not estimated	.	Not estimated
Surgical fundoplication	1.19 (0.54, 2.65)	1.28 (0.50, 3.25)	1.64 (0.58, 4.65)	Not estimated	.
Outcome: Abnormal acid exposure, RR (95% CI)					
PPI	.	1.12 (0.62, 2.02)	1.15 (0.72, 1.84)	2.23 (0.70, 7.12)	3.26 (1.29, 8.26)

Radiofrequency energy delivery	0.89 (0.50, 1.61)	.	1.03 (0.49, 2.19)	1.99 (0.54, 7.34)	2.92 (0.97, 8.78)
Endoscopic plication	0.87 (0.54, 1.38)	0.97 (0.46, 2.06)	.	1.93 (0.55, 6.76)	2.83 (1.00, 8.01)
Reinforcement of LES	0.45 (0.14, 1.43)	0.50 (0.14, 1.85)	0.52 (0.15, 1.81)	.	1.47 (0.33, 6.49)
Surgical fundoplication	0.31 (0.12, 0.78)	0.34 (0.11, 1.03)	0.35 (0.12, 1.00)	0.68 (0.15, 3.02)	.
Outcome: %Time pH < 4, MD (95% CI)					
PPI	.	0.01 (-3.57, 3.58)	0.45 (-1.88, 2.78)	0.28 (-3.02, 3.58)	2.98 (-0.11, 6.07)
Radiofrequency energy delivery	-0.01 (-3.58, 3.57)	.	0.44 (-3.82, 4.71)	0.27 (-4.60, 5.13)	2.97 (-1.76, 7.70)
Endoscopic plication	-0.45 (-2.78, 1.88)	-0.44 (-4.71, 3.82)	.	-0.17 (-3.93, 3.58)	2.53 (-1.34, 6.40)
Reinforcement of LES	-0.28 (-3.58, 3.02)	-0.27 (-5.13, 4.60)	0.17 (-3.58, 3.93)	.	2.70 (-1.82, 7.22)
Surgical fundoplication	-2.98 (-6.07, 0.11)	-2.97 (-7.70, 1.76)	-2.53 (-6.40, 1.34)	-2.70 (-7.22, 1.82)	.
Outcome: LES resting pressure, MD (95% CI)					
PPI	.	1.66 (-0.23, 3.55)	Not estimated	-0.20 (-5.68, 5.28)	-9.23 (-11.82, -6.64)
Radiofrequency energy delivery	-1.66 (-3.55, 0.23)	.	Not estimated	-1.86 (-7.66, 3.94)	-10.88 (-14.09, -7.68)
Endoscopic plication	Not estimated	Not estimated	.	Not estimated	Not estimated
Reinforcement of LES	0.20 (-5.28, 5.68)	1.86 (-3.94, 7.66)	Not estimated	.	-9.03 (-15.09, -2.96)
Surgical fundoplication	9.23 (6.64, 11.82)	10.88 (7.68, 14.09)	Not estimated	9.03 (2.96, 15.09)	.

PPI, proton pump inhibitor; GERD, gastroesophageal reflux disease; HRQL, health related quality of life questionnaire; SF-36, 36-item short form survey; LES, lower esophageal sphincter; RR, risk ratio; SMD, standardized mean difference; MD, mean difference; CI, confidence interval
