# Supplementary material for: Efficacy of Endoscopic and Surgical Treatments for Gastroesophageal Reflux Disease: A Systematic Review and Network Meta-Analysis
Source: J Pers Med. 2022 Apr 12;12(4):621. doi: 10.3390/jpm12040621 (PMC9031147; doi:10.3390/jpm12040621)
Supplement: Supplementary file 1 [file jpm-12-00621-s001.zip › 04. GERD intervention - Table S3.pdf]

Table S3. Adverse events of endoscopic or surgical treatments of GERD

Publication year, First author [reference number]	Arm 1	Arm 2	Adverse event	Death
2003, Corley [23]	Radiofrequency energy delivery	Sham + PPI	Arm 1: perforation (0%), bleeding requiring transfusion (0%), chest pain (11%), abdominal pain (3%), esophageal ulcer (3%)<br><br>Arm 2: perforation (0%), bleeding requiring transfusion (0%), chest pain (0%), abdominal pain (3%), pneumonia (3%)	None
2008, Coron [24]	Radiofrequency energy delivery	PPI	Arm 1: perforation (0%), bleeding requiring transfusion (0%), abdominal pain or discomfort (17%), swallowing pain (4%), fever (9%)<br><br>Arm 2: None	None
2010, Aziz [25]	Radiofrequency energy delivery	Sham + PPI	Arm 1: perforation (0%), bleeding requiring transfusion (0%), pneumonia (8%)<br><br>Arm 2: perforation (0%), bleeding requiring transfusion (0%)	None
2012, Arts [26]	Radiofrequency energy delivery	Sham + PPI	N/A	N/A
2017, Kalapala [27]	Radiofrequency energy delivery	Sham + PPI	N/A	N/A
2006, Montgomery [28]	Endoscopic plication	Sham + PPI	N/A	N/A
2006, Rothstein [29]	Endoscopic plication	Sham + PPI	Arm 1: perforation (0%), chest pain or discomfort (12%), abdominal pain or discomfort (9%), pharyngolaryngeal pain (9%), dysphagia (3%), pneumoperitoneum (3%)<br><br>Arm 2: perforation (0%), chest pain or discomfort (5%), abdominal pain or discomfort (0%), pharyngolaryngeal pain (10%), dysphagia (3%), pneumoperitoneum (0%)	None
2007, Schwartz [30]	Endoscopic plication	Sham + PPI	Arm 1: chest soreness (30%), abdominal pain (5%), dysphagia (50%), sore throat (40%)<br><br>Arm 2: chest soreness (0%), abdominal pain (5%), dysphagia (5%), sore throat (45%)	None
2015, Håkansson [31]	Endoscopic plication	Sham + PPI	Arm 1: abdominal pain (45%), dysphagia (18%)<br><br>Arm 2: abdominal pain (5%), dysphagia (10%)	None
2015, Hunter [32]	Endoscopic plication	Sham + PPI	Arm 1: chest pain (1%), abdominal pain (2%), dysphagia (2%), nausea (1%)<br><br>Arm 2: nausea (2%)	None
2015, Rinsma [33]	Endoscopic plication	PPI	N/A	N/A
2015, Trad [34]	Endoscopic plication	PPI	Arm 1: serious adverse event (0%)<br><br>Arm 2: serious adverse event (0%)	None

2015, Witteman [35]	Endoscopic plication	PPI	Pneumonia (5%)	1 (2%)<br>One patient died 11 months after receiving endoscopic plication. The cause of death was assumed cardiac or neurological, but the exact cause remained uncertain.
2021, Kalapala [36]	Endoscopic plication	Sham + PPI	Arm 1: major procedure-related adverse event (0%), chest pain (3%)<br>Arm 2: major procedure-related adverse event (0%)	None
2005, Devière [37]	Reinforcement of LES	Sham + PPI	Arm 1: chest or epigastric pain (69%), dysphagia (28%), fever (22%)<br>Arm 2: chest or epigastric pain (6%), dysphagia (9%), fever (0%)	None
2010, Fockens [38]	Reinforcement of LES	Sham + PPI	Arm 1: perforation (1%), bleeding (1%), chest pain (15%), epigastric pain (7%), dysphagia (9%)<br>Arm 2: perforation (0%), bleeding (2%), chest pain (7%), epigastric pain (2%), dysphagia (5%)	None
2019, Bell [39]	Reinforcement of LES	PPI	Arm 1: dysphagia (32%)<br>Arm 2: major event (0%)	None
2000, Lundell [40]	Fundoplication	PPI	N/A	N/A
2005, Mahon [41]	Laparoscopic Nissen fundoplication	PPI	Arm 1: esophageal injury (1%), hepatic injury (1%), splenic injury (2%), wrap migration (3%), respiratory infection (2%)<br>Arm 2: N/A	Arm 1: none<br>Arm 2: N/A
2006, Anvari [42]	Laparoscopic Nissen fundoplication	PPI	Arm 1: abdominal pain (4%), fever (4%)<br>Arm 2: none	None
2013, Grant [43]	Laparoscopic fundoplication	PPI	N/A	None
2016, Hatlebakk [44]	Laparoscopic fundoplication	PPI	N/A	N/A
2006, Domagk [45]	Endoscopic plication	Reinforcement of LES	Arm 1: transient abdominal pain and pharyngitis (frequency not shown)<br>Arm 2: transient retrosternal pain (frequency not shown), bleeding (4%), fever (4%)	None
2011, Svoboda [46]	Endoscopic plication	Laparoscopic Nissen fundoplication	Arm 1: perforation (3%)<br>Arm 2: abdominal pain (6%), deep vein thrombosis (6%)	None
2012, Antoniou [47]	Endoscopic plication	Laparoscopic fundoplication	N/A	N/A

PPI, proton pump inhibitor; LES, lower esophageal sphincter; N/A, not available
