# Supplementary material for: Efficacy of Endoscopic and Surgical Treatments for Gastroesophageal Reflux Disease: A Systematic Review and Network Meta-Analysis
Source: J Pers Med. 2022 Apr 12;12(4):621. doi: 10.3390/jpm12040621 (PMC9031147; doi:10.3390/jpm12040621)
Supplement: Supplementary file 1 [file jpm-12-00621-s001.zip › Supplementary Figures.pdf]

	Random sequence generation (selection bias)	Allocation concealment (selection bias)	Blinding of participants and personnel (performance bias)	Blinding of outcome assessment (detection bias)	Incomplete outcome data (attrition bias)	Selective reporting (reporting bias)	Other bias
2003, Corley	+	+	+	+	+	+	+
2008, Coron	+	+	−	+	+	+	+
2010, Aziz	?	+	+	+	+	+	+
2012, Arts	?	?	+	+	+	+	+
2017, Kalapala	−	?	+	+	+	+	+
2006, Montgomery	?	+	+	+	+	+	+
2006, Rothstein	+	+	+	+	+	+	+
2007, Schwartz	?	+	+	+	+	+	+
2015, Hunter	+	+	+	+	+	+	+
2015, Rinsma	?	?	−	+	+	+	+
2015, Trad	+	+	−	+	+	+	+
2015, Witteman	+	+	−	+	+	+	−
2017, Håkansson	+	+	+	+	+	+	+
2021, Kalapala	?	+	+	+	+	+	+
2005, Devière	+	+	+	+	+	+	+
2010, Fockens	?	?	+	+	+	+	−
2019, Bell	+	+	−	+	+	+	+
2000, Lundell	+	+	−	+	+	+	+
2005, Mahon	+	?	−	+	+	+	+
2006, Anvari	+	+	−	+	+	+	+
2013, Grant	+	+	−	+	+	+	+
2016, Hatlebakk	?	?	−	+	−	+	+
2006, Domagk	+	+	−	+	+	+	+
2011, Svoboda	+	+	−	+	+	+	+
2012, Antoniou	?	?	−	+	+	+	+

Figure S1. Risk of bias summary: the review of the authors' judgments regarding the risk of bias items for each included study.

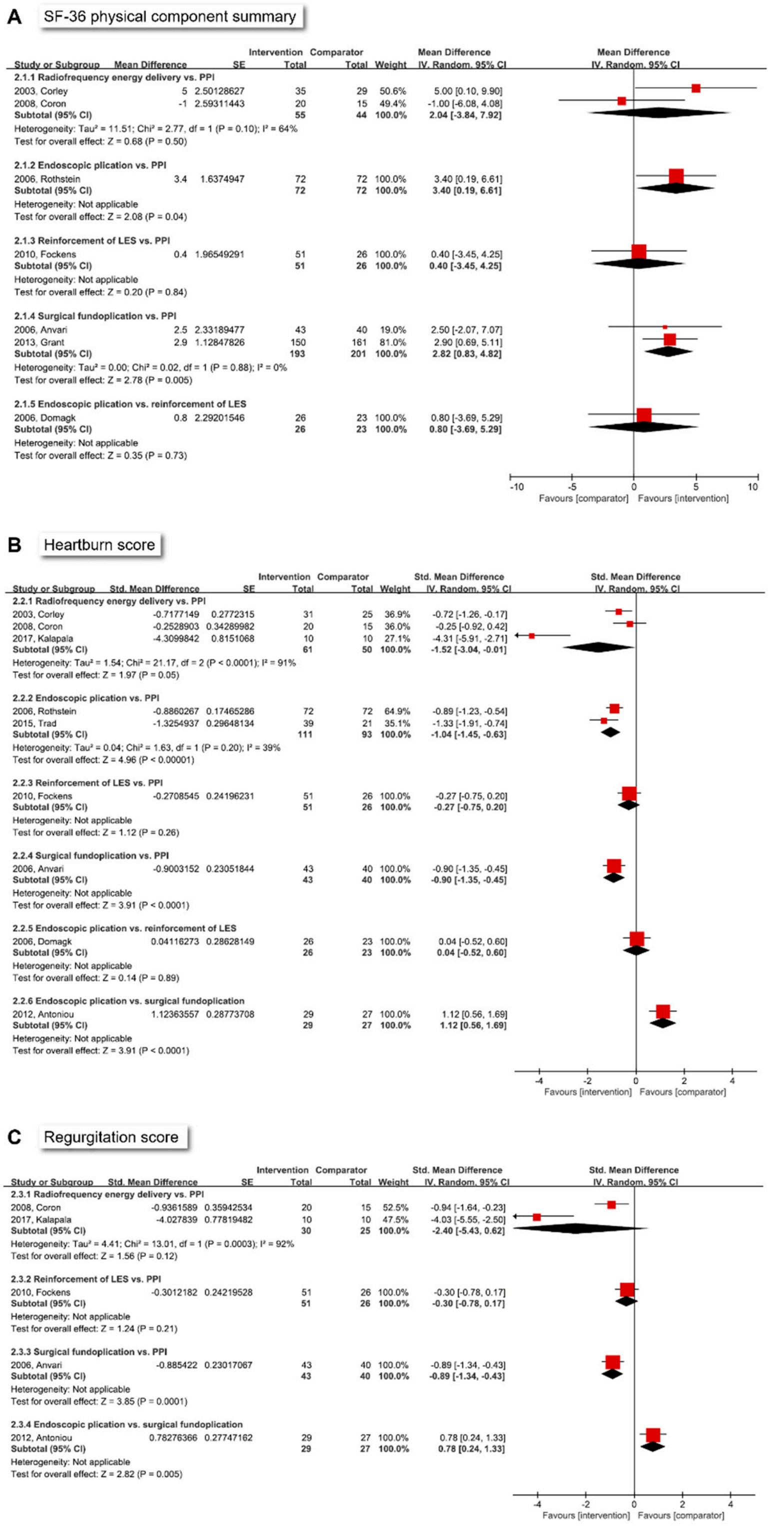

Figure S2. Direct meta-analysis of subjective outcomes of endoscopic or surgical treatments. (**A**) SF-36 physical component summary, (**B**) heartburn score, and (**C**) regurgitation score. SF-36, 36-item short-form survey; PPI, proton pump inhibitor; LES, lower esophageal sphincter; SE, standard error; IV, inverse variance; CI, confidence interval; df, degrees of freedom.

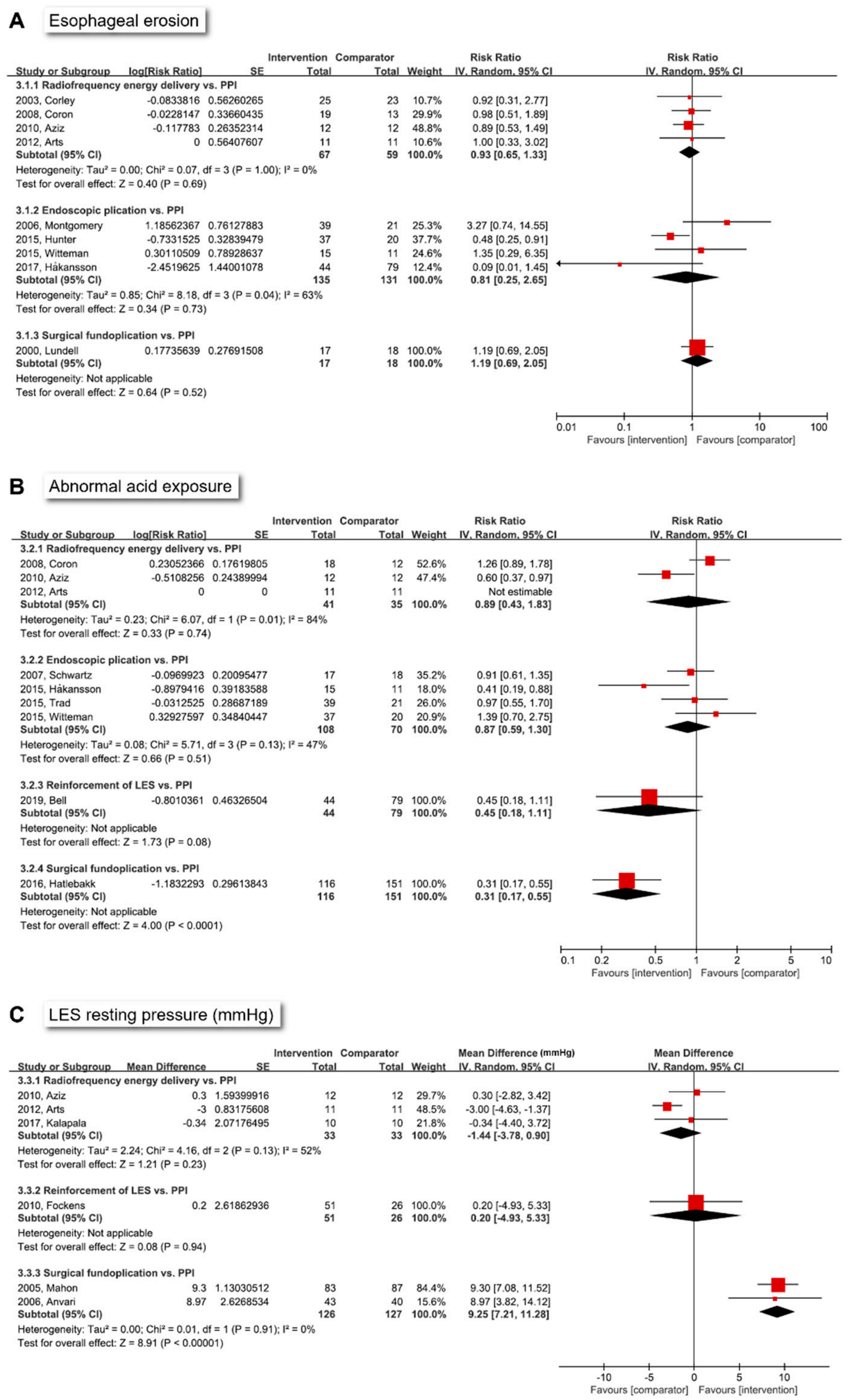

Figure S3. Direct meta-analysis of objective outcomes of endoscopic or surgical treatments. (A) Esophageal erosion, **(B)** abnormal acid exposure, and **(C)** LES resting pressure

LES, lower esophageal sphincter; PPI, proton pump inhibitor; SE, standard error; IV, inverse variance; CI, confidence interval; df, degrees of freedom.

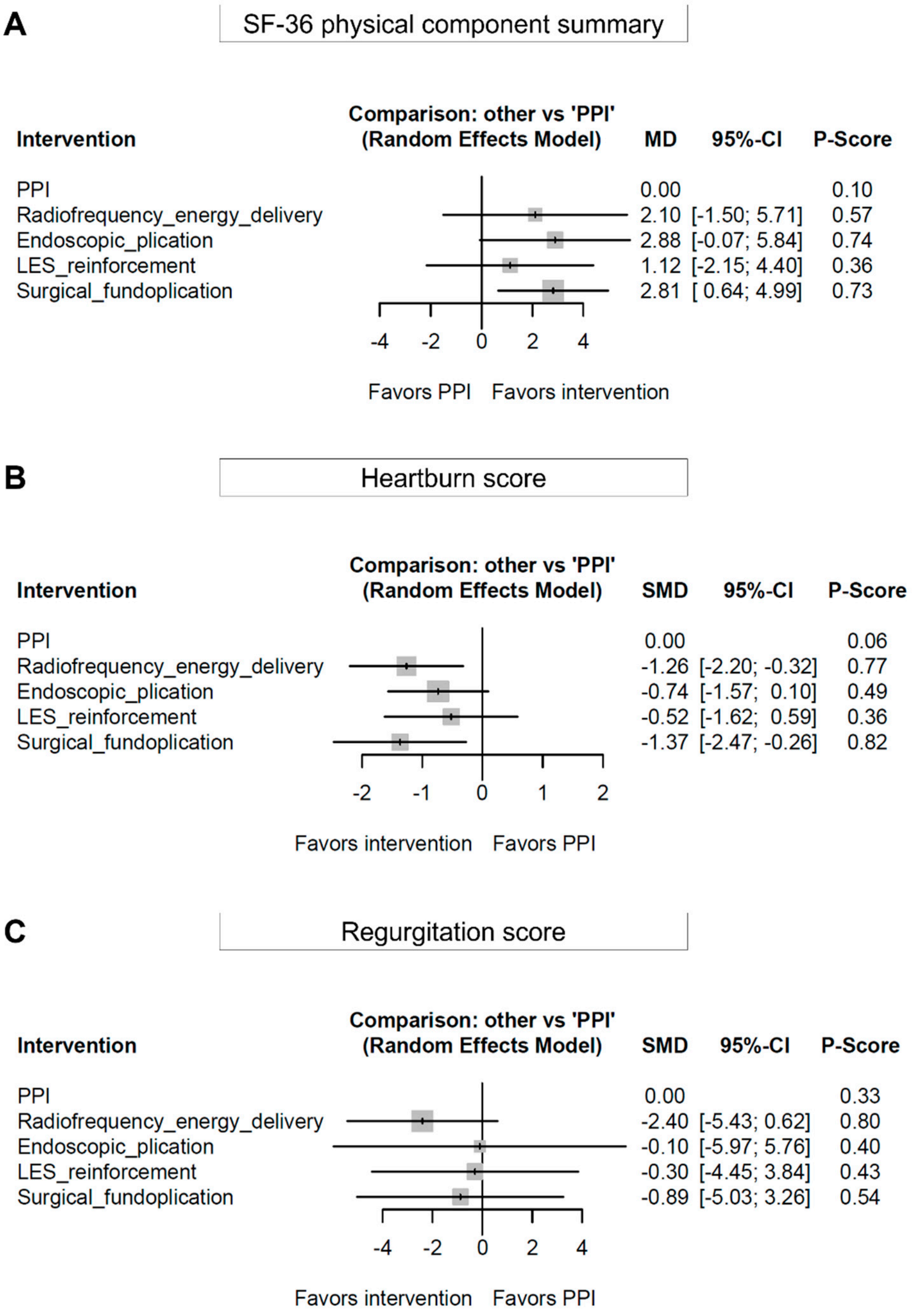

Figure S4. Comparative efficacy for subjective outcomes in the network meta-analysis. (A) SF-36 physical component summary, **(B)** heartburn score, and **(C)** regurgitation score.
The P-score indicates the mean extent of certainty that one treatment is better than another.
SF-36, 36-item short-form survey; PPI, proton pump inhibitor; LES, lower esophageal sphincter; MD, mean difference; SMD, standardized mean difference; CI, confidence interval.

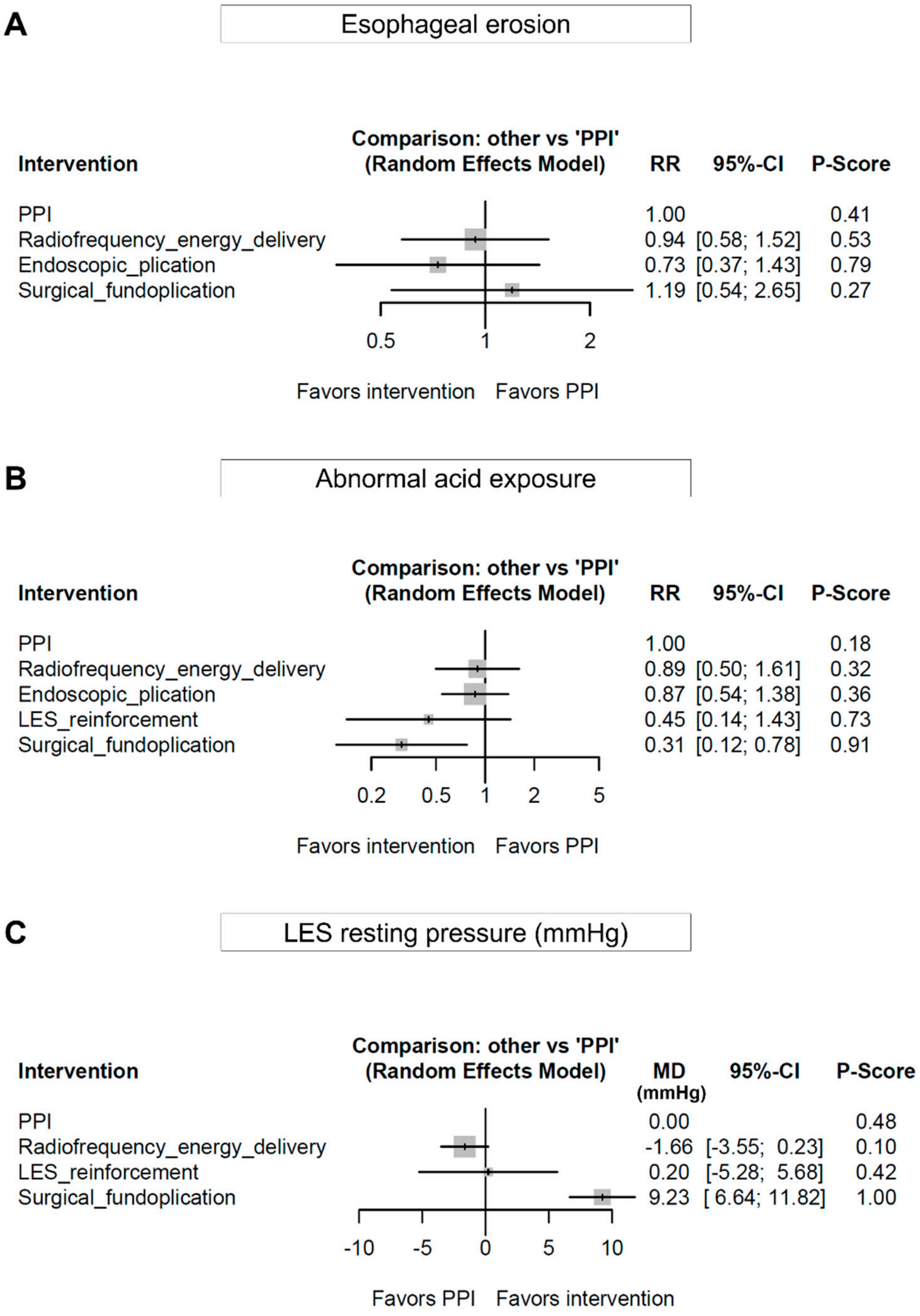

Figure S5. Comparative efficacy for objective outcomes in the network meta-analysis. (A) esophageal erosion, **(B)** abnormal acid exposure, and **(C)** LES resting pressure.

The P-score indicates the mean extent of certainty that one treatment is better than another.

LES, lower esophageal sphincter; PPI, proton pump inhibitor; RR, risk ratio; MD, mean difference; CI, confidence interval.

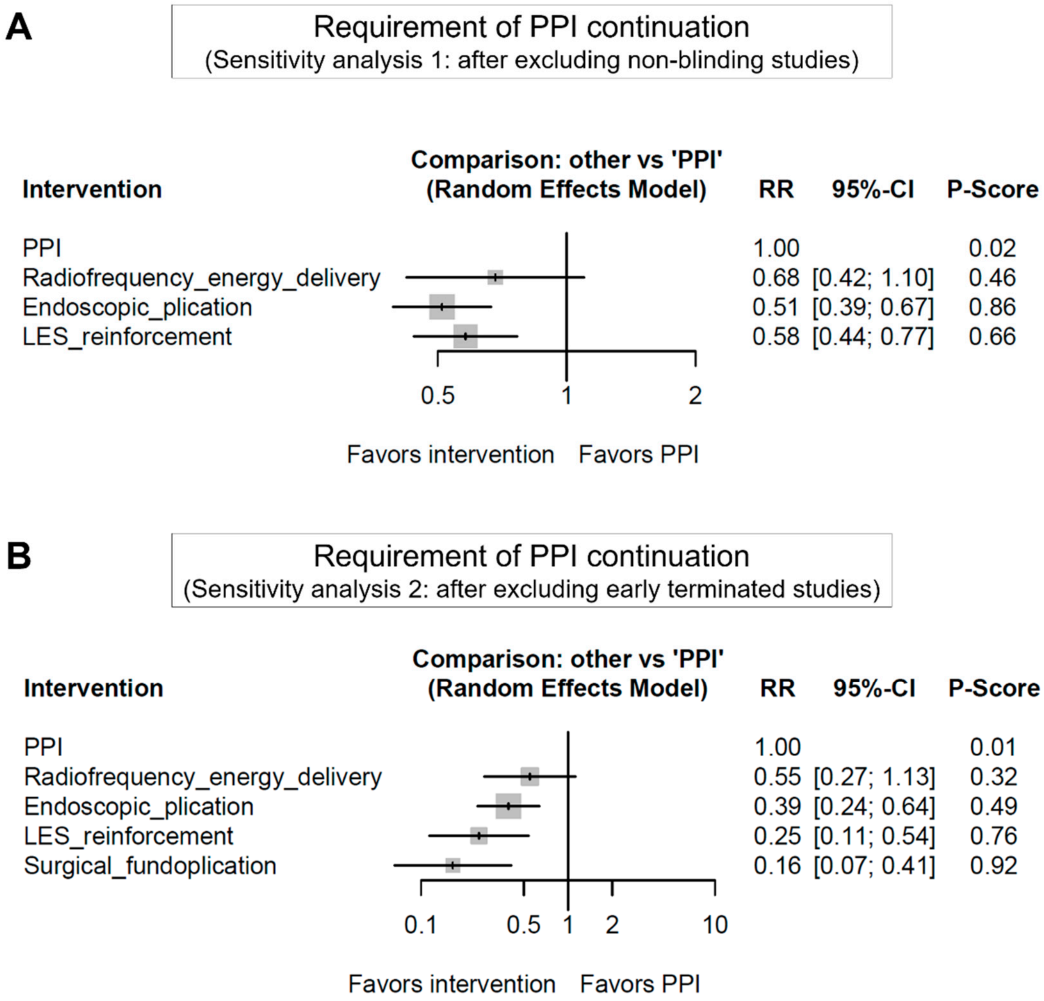

Figure S6. Sensitivity analyses of the requirement of PPI continuation. Three sensitivity analyses were performed after excluding **(A)** seven non-participant blinding studies and **(B)** two early terminated studies **(B)**. The P-score indicates the mean extent of certainty that one treatment is better than another.

PPI, proton pump inhibitor; LES, lower esophageal sphincter; RR, risk ratio; CI, confidence interval.
